# *Ageratum conyzoides* L. and Its Secondary Metabolites in the Management of Different Fungal Pathogens

**DOI:** 10.3390/molecules26102933

**Published:** 2021-05-14

**Authors:** Rubal Chahal, Arun Nanda, Esra Küpeli Akkol, Eduardo Sobarzo-Sánchez, Ashwani Arya, Deepak Kaushik, Rohit Dutt, Rashmi Bhardwaj, Md. Habibur Rahman, Vineet Mittal

**Affiliations:** 1 Department of Pharmaceutical Sciences, Maharshi Dayanand University, Rohtak 124001, India; imrubal1989@gmail.com (R.C.); aryaashwani261@gmail.com (A.A.); or deepkaushik.pharma@mdurohtak.ac.in (D.K.); 2 Department of Pharmacognosy, Faculty of Pharmacy, Gazi University, Etiler, 06330 Ankara, Turkey; esrak@gazi.edu.tr; 3 Instituto de Investigación y Postgrado, Facultad de Ciencias de la Salud, Universidad Central de Chile, Santiago 8330507, Chile; 4 Department of Organic Chemistry, Faculty of Pharmacy, University of Santiago de Compostela, 15782 Santiago de Compostela, Spain; 5 School of Medical and Allied Sciences, G.D. Goenka University, Gurugram 122103, India; rohitdatt23@rediffmail.com; 6 Centre of Medical Biotechnology, Maharshi Dayanand University, Rohtak 124001, India; bhardwajrashmi3@gmail.com; 7 Department of Pharmacy, Southeast University, Banani, Dhaka 1213, Bangladesh; pharmacisthabib@gmail.com

**Keywords:** *Ageratum conyzoides*, fungal pathogens, clinical applications, ethnomedicinal uses, precocene, toxicity

## Abstract

*Ageratum conyzoides* L. (Family—Asteraceae) is an annual aromatic invasive herb, mainly distributed over the tropical and subtropical regions of the world. It owns a reputed history of indigenous remedial uses, including as a wound dressing, an antimicrobial, and mouthwash as well as in treatment of dysentery, diarrhea, skin diseases, etc. In this review, the core idea is to present the antifungal potential of the selected medicinal plant and its secondary metabolites against different fungal pathogens. Additionally, toxicological studies (safety profile) conducted on the amazing plant *A. conyzoides* L. are discussed for the possible clinical development of this medicinal herb. Articles available from 2000 to 2020 were reviewed in detail to exhibit recent appraisals of the antifungal properties of *A. conyzoides*. Efforts were aimed at delivering evidences for the medicinal application of *A. conyzoides* by using globally recognized scientific search engines and databases so that an efficient approach for filling the lacunae in the research and development of antifungal drugs can be adopted. After analyzing the literature, it can be reported that the selected medicinal plant effectively suppressed the growth of numerous fungal species, such as *Aspergillus*, *Alternaria*, *Candida*, *Fusarium*, *Phytophthora*, and *Pythium*, owing to the presence of various secondary metabolites, particularly chromenes, terpenoids, flavonoids and coumarins. The possible mechanism of action of different secondary metabolites of the plant against fungal pathogens is also discussed briefly. However, it was found that only a few studies have been performed to demonstrate the plant’s dosage and safety profile in humans. Considered all together, *A. conyzoides* extract and its constituents may act as a promising biosource for the development of effective antifungal formulations for clinical use. However, in order to establish safety and efficacy, additional scientific research is required to explore chronic toxicological effects of *ageratum*, to determine the probability of interactions when used with different herbs, and to identify safe dosage. The particulars presented here not only bridge this gap but also furnish future research strategies for the investigators in microbiology, ethno-pharmacology, and drug discovery.

## 1. Introduction

In the modern era, the emergence and control of novel microbes poses a significant challenge to the scientific community. Various fatal diseases caused by opportunistic pathogens of different fungal strains have been reported all over the world [1,2]. Literature related to various mycological studies indicates that there has been a significant increase in the cases of candidemia in the past decade [3]. Additionally, fungal infections have affected over one billion people globally, causing more than 1.5 million deaths every year, comparable to tuberculosis deaths and more than 3 times the deaths from malaria [4]. Apart from causing mild fungal infections in healthy volunteers, a single fungal spore can start a deadly process in immune-compromised patients. Furthermore, patients suffering from chronic diseases such as diabetes, cystic fibrosis, AIDS and recurrent infection as well as patients on chemotherapy or receiving bone marrow or any other organ transplantation are at higher risk [5,6,7]. *Candida albicans*, *Cryptococcus neoformans*, *Aspergillus fumigatus*, *Fusarium*, and *Scedosporium* spp. are some of the important infection-causing fungal strains in both crops and animals. Among the two factors responsible for the increases in the number of fungal infections, one is the incessant use of broad-spectrum antibiotics, which reduces the population of non-pathogenic bacteria that usually compete with the fungi. Another worrying factor has been a rise in number of persons with lessened immune response caused by the use of chemotherapeutic drugs, immunosuppressant treatments, or by acquired immunodeficiency syndrome [8]. In the past, the various synthetic analogues of imidazoles and azole nucleus were developed to treat fungal infections. However, with passage of time, their effectiveness has diminished; additionally, there is the chance that the infection will relapse after their use [9]. Moreover, the currently established antifungal candidates suffer from a number of limitations that render their use problematic, such as amphotericin B-linked dose-limiting nephrotoxicity, quick resistance development associated with flucytosine, interactions among drugs, and resistance development against azoles. Keeping in view the above facts, we can say that there is an urgent need to search for new and effective antifungals [10].

Herbal medicinal resources are deliberated as being a vital part of nature. For many years, novel active constituents, particularly those of natural origin, have been of great interest to researchers owing to their exclusive chemical frames and potent bioactivities. The importance of plant actives in the development of novel drugs can be judged by the fact that most of the therapeutic agents approved in last century were derived from plants or natural sources [11]. In light of above, we can say that natural compounds can be effectively used in the prevention and treatment of various infections. Additionally, a vast number of extracts or secondary metabolites such as terpenoids from essential oils, alkaloids, flavonoids, etc. have been effectively established as antifungals [9]. Preferably, new-fangled herbal fungicides should contribute improvements in target specificity, broad-spectrum activity, diverse action mechanisms, and the lack of cross-resistance with regard to the presently offered synthetic options. Many allelopathic weeds’ oils and extracts have been reported in the literature as potential substitutes for menacing synthetic fungicides [12,13,14].

It is evident from previous research findings that antifungal compounds from *Ageratum conyzoides* L. can be approached for the development of safer and more economically and ecologically sound substitutes for the presently established fungicides. *A. conyzoides* L. is an aromatic herb possessing an extensive history of benefits in traditional medicine around the world. A variety of secondary metabolites belonging to different chemical classes such as flavonoids, alkaloids, chromene, terpenoids, coumarins, and sterols from *A. conyzoides* have been isolated and characterized. Furthermore, investigations have also reported the presence of a vast variety of phytoconstituents in the herb’s essential oil, such as benzofurans (precocene I, precocene II, and ageratochromene dimer), coumarin, chromene, flavonoids (kaempferol, quercetin, quercetin-3-rhamnopiranoside), alkaloids (caffeic acid, echinatine, phytol, and pyrrolizidine alkaloids), sterols (stigmasterol, β-sitosterol, and friedeline), terpenes (α-pinene, β-pinene), and eugenol varying in their concentrations from one place to another. These secondary metabolites are claimed to have diverse medicinal properties, including radioactive, antidiabetic, antimicrobial, anti-inflammatory, antioxidant, anticancer, and wound healing properties and many more [15,16,17,18].

Along with the chemical content and pharmacological profile the researchers have also explored the allelopathy and invasiveness of *A. conyzoides* [19,20]. However, not all aspects related to the potential use of this medicinal plant against various fungal pathogens have been comprehensively reviewed to date. Therefore, in order to ascertain the safe grounds for the further application of the plant at commercial scale, the present review, aims to present the ignored antifungal potential of *A. conyzoides*, along with an overview of the safety studies conducted on the plant.

## 2. Methodology

The intellectual facts necessary for this comprehensive study were achieved by carrying out an up-to-date search (2000–2021) using several globally recognized scientific databases, such as Science Direct (http://www.sciencedirect.com, accessed in July 2020), Directory of Open Access Journal (DOAJ), Scopus (http://www.scopus.com, accessed in July 2020), PubMed (http://ncbi.nlm.nih.gov/pubmed, accessed in August 2020), SpringerLink (http://www.spinger.co.in, accessed in August 2020), Google Scholar (http://www.onlinelibrary.wiley.com, accessed in September 2020), Web of Science Core, recognized books, thesis, and abstracted and non-indexed and non-impacted journals. Searches were conducted without imposing any language restrictions and using keywords: “*A. conyzoides*“ and/or paired with “antifungal”, “natural”, “precocene II”, and “phytochemistry”. The current review highlights the traditional uses of the various parts of *A. conyzoides* across the world, the activity of the different extracts or oil obtained from the plant against important fungal strains, together with either acute or chronic toxicological studies performed on the plant to frame its safety for humans.

## 3. Taxonomy, Description and Botanical Characteristics

### 3.1. Taxonomical Classification

**Figure 1:**
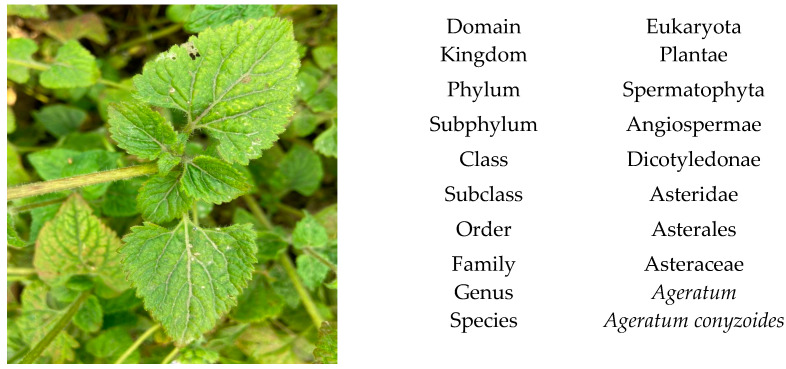
*Ageratum conyzoides* L. (Picture was taken in the month of April from the agricultural land in Jammu, India.).

The *A. conyzoides* (Figure 1) and its taxonomical classification was validated through a standard database [21].

### 3.2. Description

*Ageratum conyzoides* (Billy goat weed) is typically known as Jangli pudina or appa grass in Hindi, Mentrasto in Portuguese, Mejorana in Spanish, and Igbo in West Africa [22]. The word “*Ageratum*” originated from the Greek word \”a geras\”, which means “non-aging” and speaks to the long life of the entire herb; because of its resemblance to the plant *Inula helenium*, (Greek—“kónyz,”) the particular appellation “*conyzoides*” is given [23,24]. Its name “billy goat weed” or “goat weed” is attributed to its odd odor, which is similar to that of the male Australian goat. This herb is generally distributed in tropical America—Florida and Caribbean—and also scattered in West Africa and Southeast Asia, India, China, and Australia [25].According to Das and Mukherjee [26], the genus *Ageratum* hosts about 40 species overall, but only two of them as exotic weeds are reported in India: *A. conyzoides* and *A. houstonianum*. Studies of *A. conyzoides* have provided evidence of the presence of a wide variety of phytochemicals, such as alkaloids, tannins, terpenoids, chromenes, coumarin, flavonoids, saponins, glycosides, phenols, and resins [27,28,29,30,31], along with other nutrients such ascertain amino acids (essential and non-essential) [32], vitamins (A, B, B_2_, B_6_, C, E, thiamin, and niacin), carbohydrates, and their reducing forms [33].

### 3.3. Botanical Characteristics

*A. conyzoides* is a tropical, annual, straight (attaining a height of approximately 1 m), hairy, slender, branched, and malodorous annual herb. It has yellowish brown, shallow, fasciculate, and fibrous roots that are fixed weakly to soil. The stem is aerial, cylindrical, green, and week in younger plants but with time turns to slightly brown and strong [34]. Leaves are simple, opposite, stalked oval shaped, 5–50 mm wide, and 20–100 mm long, covered with white fine hairs, with prominent veins, and an attenuated base with acute tip and toothed margin. The branched and terminal inflorescence bears about 4–18 flower heads (60–70 individual flowers) that are white, purple, or light blue in color, carried by long peduncles (50–150 mm long; 5 mm across) and fenced by two/three oblong, green bracts. The straight petiole shows convex-concave contour. The fruits are sharply angled black achenes, coarsely hairy, bearing five, hardly six, pappus, white to cream in color with upward spines [23,35].The herb is scattered in India at full length along with the Himalaya region and typically cultivates on abandoned, cultivable, or devastated spots. Due to its broad adaptability toward varying ecological conditions, its sustainable reproduction prospective, and its allelopathic behavior, the plant grows untroubled and is hard to eradicate [25]. The plant is positively photoblastic, where seeds germinate with light and usually lose their viability within a year if buried under the soil [24].

## 4. Ethnopharmacology

For ages, this nuisance weed has been recognized globally for its healing power and has been well acknowledged for its insecticidal and pharmacological activities [36,37]. The wound healing potential of its crude extract was found more imposing even than Vaseline gauze [38]. Every single part of the plant has some remedial significance. An article by Yadav et al. [36] indicated its benefits in the treatment of various ailments; for instance, the leaves for headache, pneumonia, malarial and typhoid fever, leucorrhea, sore gums, and uterine and throat infections; the roots for infant diarrhea, lithiasis, and as an antitumor treatment; and the flower buds for sleeping sickness and vermifuge, as a tonic, as an anti-itch and antitussive treatment, and for killing lice. Here, some traditional uses of the plant around the world are outlined in Table 1.

In Ayurveda, an ancient remedy system that originated in India, this plant holds an amazing place and is used for fomentation in leprosy [61] and in treating pediculosis [62]. Furthermore, it is one of the proud remedies used traditionally for prostate complaints and venereal diseases [63]. Other indigenous practices of the world define the plant as an anti-diabetic [64], anti-inflammatory, and anti-microbial [65] treatment, curing diseases such as cephalgia, dyspnea, enteralgia, fever [66], malaria [67,68], spasm [33], and pneumonia [69], as well as use as an insecticidal [70]. Studies evince various prevalent traditional uses [71,72] interfused along with modern biological potential of this remarkable plant, such as use as an anti-convulsant [73], acaricidal [74], nematocidal, hypoglycemic or antiglycemic, antioxidant [75,76,77], anticoccidial [78], and as a cure for urolithiasis [79]. Many ethno-botanical surveys confirm the use of the plant in the treatment and management of HIV/AIDS [39,80].

In addition to medical applications, *A. conyzoides* also possesses more or less superstitious and mystical aspects. For example, it is believed in Ivory Coast that the plant shields devotees of a snake sect from snakebite and also from bad spirits and demons if used in combination with other plants. In Yoruba, it is used to placate witches; in Gabon, it is used as a part of sophisticated sorcery [47]; and in Congo, leaf sap on hands is supposed to bring good luck to card players. Moreover, it is a common perception there that, when rubbed with sap and pricked with a needle on the hand, the accused will feel a pain only if they are guilty [16].

## 5. Pathogenic Fungal Strains vs. Antifungal Constituents Isolated from *Ageratum conyzoides*

Fungal diseases are a global problem and are regarded as a considerable threat not only to humans but also to vegetation growth and storage, rendering foodstuffs inappropriate for human intake by retarding their nutritional value and at times by releasing mycotoxins. *Fusarium*, *Aspergillus*, and *Penicillium* are some genera guilty of producing the most concerning toxins of major field crops, including oilseeds and cereals. These mycotoxins such as aflatoxin B_1_, ochratoxin A, and fumonisin B_1_, are liable to affect humans and animal health adversely by encouraging teratogenicity, mutagenicity, and hepatotoxicity, resulting in oedema, immunosuppression, hepatitis, hemorrhage, hepatic carcinoma, kidney failure, and esophageal cancer [81]. Despite its negative economic and environmental impact, species *Ageratum* holds a distinct place among the natural resources being exploited for their antifungal properties because of the presence of an extensive range of secondary metabolites such as flavonoids, chromenes, chromones, coumarin, benzofurans, terpenoids, steroids, and alkaloids. The plant is capable of demonstrating its pesticidal, fumigant, antifungal, and antimicrobial properties, making it a noteworthy substitute for current pathogen-regulating measures. A substantial literature favors the plant by providing the experimental rationale in support of the antifungal activities associated with this invasive plant [82,83].

**Figure 2:**
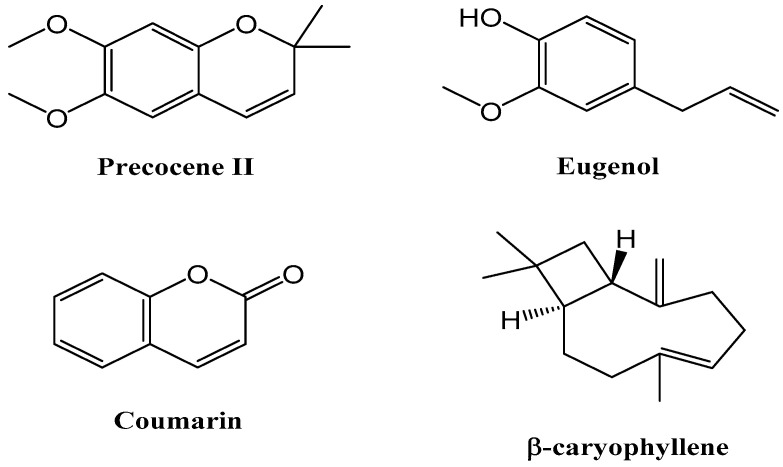
Chemical constituents of *Ageratum conyzoides* with antifungal activity.

Many compounds from the plant have been isolated, characterized, and evaluated for their fungicidal or fungistatic properties, which are more systemic, less phytotoxic, and more eco-friendly. Focal antifungal constituents identified and isolated (Figure 2) from the plant are precocene II, coumarin, β-caryophyllene, and eugenol, whose broad spectrum fungitoxic behavior is marked by an immense literature (Table 2). However, the results of the studies in the context of their constituent composition and antifungal activity are greatly influenced by (a) geo-ecological variations, i.e., altitude, latitude, and average temperatures; (b) the part of the plant that is used, such as the whole plant, inflorescence, roots, shoots, or leaves; (c) the time of collecting sample (January, April, December, September) or post-harvest; (d) extract type/solvent taken (essential oil, semisolid, dried/ethanol, aqueous, acetone, ether, or dichloromethane fractions etc.); and (e) the fungal strain chosen for the investigation [84,85,86,87,88,89]. Furthermore, upon exposure to various stress factors, the plant was found to release more allelochemicals, such as ageratochromene and its analogues, flavones, sesquiterpenes, and monoterpenes [90].

Various extracts and essential oils obtained from the plant have been reported to possess their action against pathological strains either by destroying fungal cells or by preventing cell growth and reproduction. Together, these constituents are also capable of obstructing the release of mycotoxins, including various sorts of aflatoxins. Inferences of various studies affirming the antifungal activities related to the plant are conferred below, validating the astute use of *A. conyzoides* in the biological management of plant and animal pathogenic fungi. In addition, this is the first review of this kind where effort has been put forth to aggregate investigations conducted on the plant concerning its antifungal prospects and toxicological standing.

### 5.1. Ageratum conyzoides against Fungal Genus Aspergillus

Among the most concerning fungal pathogens having multifarious properties, virulent genera *Aspergillus* dominates. As a consequence of its ubiquitous and aflatoxin-releasing nature along with its capability to colonize itself on a range of eatables, this species has grabbed a momentous pull of the attention of the world’s scientists. These microbes hold certain virulence factors that enable them to spread a distressing amount of damage to animals and plants. Fungal pathogens cause significant harvest losses in agriculture, spread disease in animals, and lead to life-threatening mycosis in humans; immune compromised individuals suffer a particularly high mortality risk [88,104]. To restrain these diseases and pests, some researchers have struggled to develop new eco-friendly alternatives to conventionally used synthetic approaches. Among the plants screened, many plant extracts, including *Ageratum*, have proved their efficacy and safety when validated and verified for their antifungal potential.

Widodo et al. [105] screened various fractions of *A. conyzoides* with different solvents, and out of those, crude ethanolic extract showed agreeable activity against various fungal strains such as *A. niger*, *C. albicans*, *M. gypseum*, and *T. mentagrophytes*, whilst less activity was reported against bacterial strains such as *Pseudomonas aeruginosa*, *E. coli*, and *Staphylococcus aureus*. A white spike crystalline compound was also isolated successfully from the acetone fraction of *A. conyzoides* leaves, which was characterized as a coumarin, and this derived compound proved its better fungicidal activity (72 h clear inhibition zone) in comparison with the standard miconazole nitrate treatment (24 h inhibition zone) against *A. niger*, launching it as a novel antifungal alternative. Some other authors have also assessed the plant invitro for *Aspergillus* growth inhibition and supported the extract’s activity against the fungus. Wuyep et al. [88] aimed to evaluate the aqueous and ethanolic extracts of *A. conyzoides* against various strains of *Aspergillus*, *A. niger*, *A. fumigatus*, *A. ustus*, *A. terreus*, and *A. tamarii*—strains that are frequently involved in animal and plant fungal diseases. Extracts were assessed quantitatively in vitro with the well diffusion technique, then they were challenged and compared with isolated test standard and controls, respectively. Aqueous extract possessed a higher percentage yield as well as greater antifungal activity in comparison withthe ethanolic extract. The lowest antifungal activity was shown against *A. fumigatus* while the maximum activity was recorded against *A. ustus* and *A. tamarii* with the respective inhibition zones of 8.0 ± 0.1 mm, 20.0 ± 0.6 mm, and 15 ± 0.3 at a concentration of 800 mg/mL. However, in vivo activity of the plant extracts using the standard organisms did not correlate thoroughly with the in vitro tests. Not only could the fungus be controlled by the use of herbal fungicides, but also the carcinogenic and toxic metabolites and aflatoxins released by these fungi could also be controlled, particularly those from *Aspergillus parasiticus* and *A. flavus*. Every year, mycotoxins contaminate almost 25% of the world’s total food commodities. The anti-aflatoxigenic activity of *Ageratum* essential oil was investigated via poisonous-medium method by using different concentrations of the obtained oil, i.e., 1500, 1000, and 500 ppm. The essential oil completely inhibited *A. parasiticus* growth and repressed >84% of the aflatoxin production at the concentrations of 0.75 mg/mL and 0.5 mg/mL, as demonstrated by tandem mass spectrometric analysis. Additionally, the volatiles from *A. conyzoides* green leaf tissue were reported to hold fumigant activity against *A. parasiticus* [106].

Adjou et al. [107] recommended replacing harmful chemicals with natural antifungals and provided evidence in favor of essential oil obtained from the hydro-distillation of *Ageratum* leaves. *A. flavus* (La3228) and *A. parasiticus* (Ab2242) fungal growth and aflatoxin synthesis were inhibited when exposed to the essential oil. Minimal fungicidal concentrations (MFCs) reported were 3.0 μL/mL and 2.5 μL/mL against *A. parasiticus* (Ab2242) and *A. flavus* (La3228). Minimal inhibitory concentrations (MICs) recorded were 2.5 μL/mL and 2.0μL/mL for *A. parasiticus* (Ab2242) and *A. flavus* (La3228), respectively, whilst the key components analyzed were precocene II, precocene I, cumarine, and trans-caryophyllene. The fungal inhibition zone was evaluated by disc diffusion method and compared with positive control, nystatin. From the studies, it was revealed that this virulent species is very susceptible to a chromene isolated from the plant: precocene II (6,7-dimethoxy-2,2-dimethyl-2-chromene). Some investigations witnessed the potential of precocene-II against the pathogenic genus *Aspergillus*. Precocene II (46.35%), precocene I (42.78%), cumarine (5.01%), and trans-caryophyllene (3.02%) were the main constituents identified by Nogueira et al. [92] in the essential oil obtained from the leaves distillation of *A. conyzoides*. *Aspergillus flavus* growth (maximum 63% at 1 μg/mL) and aflatoxin biosynthesis (100% at 0.10 μg/mL) were inhibited when treated with the oil by causing irreversible structural changes to the fungal mitochondria. As expected, similar composition showed similar activity because biological performance is correlated with the existence of secondary metabolites. This hypothesis was later confirmed by Esper et al. [94] when investigation was carried out about the variation in chemical composition and antifungal effect of the essential oils obtained from three different locations (Ribeirão Pires, Ibiúna, and Campinas) of São Paulo state, Brazil. Precocene I and II were in higher proportions in the oil from Ribeirão Pires (1) and Ibiúna (2), while in the essential oil obtained from the leaves collected from Campinas (3), precocene I, (*E*)-caryophyllene, and α-humulene were in highest ratio as compared with traces of precocene II. The percentage of fungal *A. flavus* growth inhibition was 64% and 60% by oil (1) and (2), respectively, whilst the oil (3) was inactive. Alternatively, all the three oils inhibited the fungus sporulation for >120 days, which may be attributed to the synergism of the oil’s constituents. In this way, the plant has shown its potential for controlling not only fungal growth but also aflatoxin production, which is vital because consumption of toxin-affected food commodities may cause intensive aflatoxicosis in human beings. These studies also provoked the curiosity of researchers toward precocene II, urging them to explore oil’s potential against fungal infections.

### 5.2. Ageratum conyzoides against Genus Fusarium

*Fusarium* species is one of the economically crucial pathogenic fungal groups that is responsible for triggering many diseases in plants, for instance, head blight, cereal grains scab, vascular wilt (fruit rot), and crown rot disease, whilst the fungi occasionally affect animals. In human beings, this group is found to carry a broad range of infections from superficial (onychomycosis and keratitis) or localized invasive infections to disseminated ones (affecting exclusively immune-compromised individuals) [108]. Several *Fusarium* species in humans have appeared as imperative opportunistic pathogens that can cause hyalohyphomycosis specifically in patients undergoing bone marrow transplantation or in those who are burn victims. Pathogenic species of *Fusarium* are very hard to control or treat because of their resistant nature and their ability to survive for a long time in soil with or without the presence of a host plant. Often the species is isolated from the human cornea and rarely from skin, nail, tissue, pleural fluid, urine, or blood [109]. Synthetic agents that are used to curb these pests and related diseases have some associated hazards, such as toxicity in non-target animals and decrease in crop productivity.

Therefore, in the modern world, biological control strategies are gaining increasing importance worldwide. When different pathogenic microorganisms including *Fusarium* were exposed to different extracts of *A. conyzoides*, the pathogenic microorganisms were found to be suppressed in most of the studies, proving the fungicidal rationale for *A. conyzoides*.

The literature documents a few but very convincing reports stating the antifungal activity of *A. conyzoides* extracts against fusarium species. Rai and Acharya [110] screened 11 species of the plants belonging to the family Asteraceae for their antimycotic potential using the disc diffusion technique and listed *A. conyzoides* within those reported to effectively inhibit mycelia growth of *Fusarium oxysporium*. However, the higher positions were occupied by various other oils obtained from the other plants of same family, such as *Tagetes erecta* and *T. patula*. Adekunle [111] stated that ethanolic *A. conyzoides* extracts efficiently inhibited all the eight fungi tested, including *Fusarium solani* and *Candida albicans*, in comparison with nine other plant extracts examined. Aqueous counterparts of the extracts were also studied against the ethanolic ones but were found to be less effective. Sidra and Uzma [112] evaluated *n*-hexane, methanolic, and aqueous extracts of the different parts of the weed *A. conyzoides* (root, leaf, stem, and inflorescence) against *Fusarium solani* Mart. (Sacc.), isolated from the roots of eggplant. When fungus was exposed to varying concentrations (2, 4, and 6% *w*/*v*) of different extracts, its growth was reported significantly suppressed. For control over fungal biomass, the observed order of activity followed for the plant part used was leaf > inflorescence > stem > root, and for the solvents it was *n*-hexane > methanolic > aqueous. The study demonstrated that all plant parts had great allelopathic potential and extracts were fungitoxic to the wilt pathogen *F. solani*. This ability of different extracts to effectively reduce fungal biomass growth shows that they can be used in the management of various diseases. Later, the study of Ilondu [113] was also in line with the earlier findings of Sidra and Uzma [112]. Varying concentrations (8–120 mg/mL) of ethanolic leaf extract of the plant *A. conyzoides* were evaluated for their antifungal activity against leaf spot fungi, *Fusarium lateritium*, *F. Solani*, and *Cochliobolus lunatus* by using the poisoned food method. The phytochemical analysis of the extract showed the presence of terpenes and alkaloid in moderate concentration, which may be attributed to the fungicidal effect of the plant. Inhibition potential of the extracts was significant (*p* < 0.05) and concentration dependent. Minimum inhibitory concentrations (MICs), where no fungal growth was reported, were 120, 80, and 88 mg/mL for *F. lateritum*, *F. solani*, and *C. lunatus*, respectively. These results are encouraging steps toward the development of natural and less expensive fungicides for the management of several diseases caused by Fusarium species.

### 5.3. Ageratum conyzoides against Candida Pathogen

Among the main sources of nosocomial septicity, *C. albicans*, a dimorphic fortune-hunter microbe, holds the fourth position. *Candida* is generally found in the intestine and in the mouth and in very small amounts on the skin, none of which is problematic. The healthy bacteria of the body keep a check on the *Candida* level, but in conditions involving disruption of healthy bacterial levels or a less than competent immune system, this fungus can show uncontrolled growth called candidiasis. The fungus can be easily isolated from cancer patients receiving chemotherapy or from the patients with diabetes or HIV/AIDS. Fungal biofilms are resilient toward a number of antifungal agents, making traditionally used fungitoxic drugs ineffective in the management of candida fungal infections [114]. Flaws in diagnostic techniques and a comparatively smaller collection of antifungal agents make the situation more stressful and justify the necessity for newer herbal antifungals.

**Figure 3:**
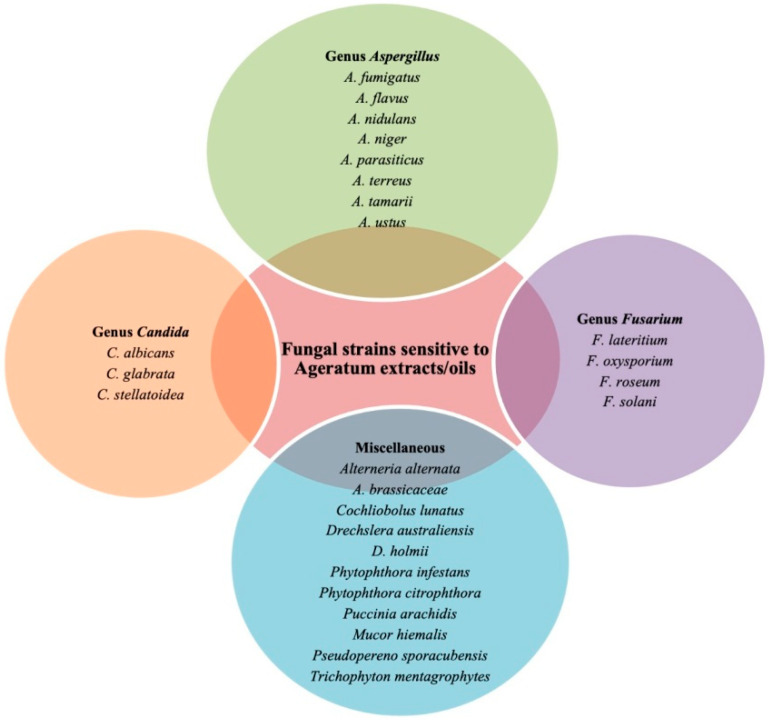
Various pathogenic fungal strains sensitive to *Ageratum* extracts/oils.

Hoffman et al. [46] found *n*-hexane extracts of *Ageratum* remarkably active against both of the tested fungi: *C. albicans* and *A. fumigatus.* Their zones of inhibition were observed to be 25.4 mm and 42.1 mm, respectively, similarly competent to the standard antibiotic counterpart, fluconazole. Later, the search for inexpensive and non-resistant antifungals led Osho and Adetunji [85] to inspect the essential oil of *A. conyzoides* stem, leaves, and roots parts for their antimicrobial activities. The sensitivity of *Candida* species (*C. glabrata*, *C. stellatoidea*, and *C. albicans*) was determined through the well diffusion procedure and MIC. At 10 μL/mL concentration, essential oil from *A. conyzoides* leaves was found most effective against the tested fungus, *Candida stellatoidea* (19 ± 0.4 mm inhibition zone), whereas essential oil from the stems expressed little or no activity against *C. glabrata*. Minimum inhibitory concentrations ranged from 2 mg/mL to 4 mg/mL, suggesting the use of the plant in controlling infections caused by the tested strains of *Candida* (*C. stellatoidea*). Recently, Khastini et al. [54] assessed the aqueous and ethanolic extracts obtained from the maceration of *Ageratum* leaves for their antifungal activity, together with chemical composition screening and percentage yield calculation. Individual extracts evaluated by the disk diffusion technique were found to inhibit the *C. albicans* growth significantly because of the presence of various secondary metabolites in the extracts, such as flavonoids, alkaloids, tannins, steroids, and saponins and offered the same minimum inhibitory concentration (MIC) of 80 mg/mL. However, the diameter of inhibition zone was different: 1.59 mm and 1.55 mm for ethanolic and water fractions, respectively. Results confirmed that both the extracts were comparatively weaker fungicides than the ketoconazole that was used as a positive control. Various fungal strains suppressed by the selected plant extracts or oils are depicted in Figure 3.

## 6. Mechanism of Action (MOA)

**Figure 4:**
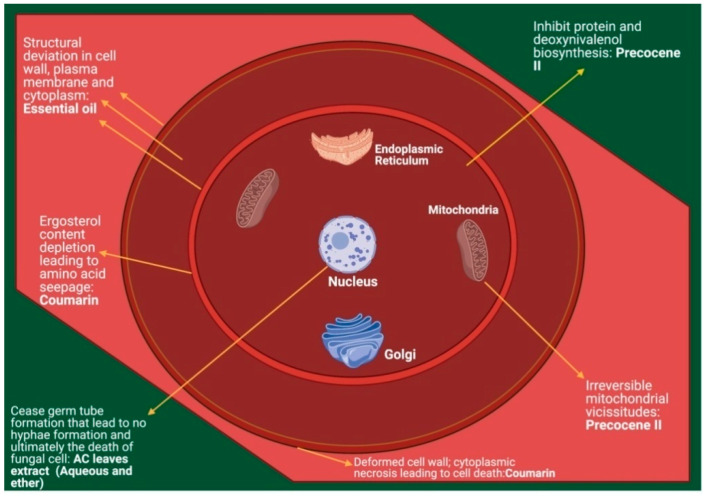
Effect of various *Ageratum* oil/constituents/extracts on fungal cell morphology.

Various extracts and essential oil obtained from the *A. conyzoides* plant have been screened to identify the molecular mechanisms underlying their antifungal activity or how these compounds target pathogenic microbes. Chief antifungal compounds isolated from *A. conyzoides* were effective against a number of obsessive strains either by precluding cell growth and reproduction or by eradicating the fungal cells directly (Figure 4). In conjunction, these compounds can also proficiently obstruct the discharge of toxic mycotoxins. Inhibition of various fungi by diverse mechanisms also satisfies the requisite aptitude needed for the development of novel anti-resistant antifungals. Khastini et al. [54] observed that aqueous and ether extracts of *Ageratum* leaves inhibited fungal growth by halting the formation of germ tubes by spores in the presence of the tested fungi, which is crucial for the microorganism’s survival because new hyphae formation can only begin with the germ tubes. Studies substantiating the MOA of precocene II conclude that the compound exhibits its activity either by retarding fungal growth or by stopping the release of mycotoxins such as aflatoxins (B_1_, B_2_, G_1_ and G_2_) and trichothecenes. Yaguchi et al. [115] suggested that precocene II can inhibit deoxynivalenol biosynthesis, a contaminant released by *F. graminearum* that reduces grain utilization. When analyzed for its mechanism of action, precocene II was found to reduce the mRNA intensities of encoding proteins requisite for deoxynivalenol biosynthesis without inhibiting fungal growth. In their investigations of the fungus *A. flavus*, Nogueira et al. [92] showed that variable concentrations of *A. conyzoides* essential oil checked mycelial growth at different magnitudes. However, at 0.10 μL/mL concentration, aflatoxin B_1_ production was withdrawn completely. TEM (transmission electron microscopy) analysis revealed ultra-structural deviations in the cytoplasm and cell wall of fungal cells treated with various oil concentrations in comparison with untreated cells. The molecular mechanism involved the destruction and deletion of electron-dense granules (EDGs) from fungal cells, whereas the plasma membrane became villiform and rough and sometimes decoupled with the cell wall. Mitochondrial cristae ridge polarization experienced a decline, which led to disruption of the internal structure of the organelle. Predominantly, mitochondria and plasma membrane swallowed the pathologic alterations and suffered irreversible morphological vicissitudes.

Later, with the help of affinity magnetic bead technique and recognized a protein of mitochondrial outer membrane, Furukawa et al. [93] explored the molecular progression by which precocene II tended to inhibit trichothecene fabrication in *F. graminearum*, with the voltage-dependent anion channel (VDAC) as the precocene II-interacting protein. Precocene II escalated the mitochondrial proteins (oxidized) concentration and superoxide level in mitochondria, which led to the discontinuation of trichothecene production in the key causal agent of *Fusarium* head blight and trichothecene contamination in grains. Subsequently, several other researchers also buoyed the influence of precocene II in disturbing the cell structure and mitochondria of fungi, proving it to be a progressive antifungal agent. Thati et al. [116] studied the mechanism by which some silver-coumarin complexes exhibited their antifungal potential against *C. albicans*, a pathogenic strain. Results demonstrated a disruption of respiratory function and depletion of the ergosterol content of the fungal cell, which resulted in a deformed plasma membrane because membrane integrity and membrane fluidity are regulated by ergosterol in fungal cells. This sequence of processes enhanced the trans-membrane seepage of the amino acids, leading finally to apoptosis. These upshots were further supported by the findings of Widodo et al. [102], who performed scanning electron microscope (SEM) and transmission electron microscope (TEM) analysis to study the influence of coumarin on *C. albicans* that was isolated from *A. conyzoides*. This exploration confirmed that the compound damaged fungal cells by pores development in the cell wall, allowing the escape and necrosis of cytoplasmic content, leading to death. Remarkably, the anti-candida mechanism reported for coumarin was different from other antifungal extracts obtained from medicinal plants such as *Swietenia mahogany* and *Barleria grandiflora* [117,118].

Having been established on the grounds of constructive deliberations, the recommendation of extracts and essential oils from *A. conyzoides* as novel plant-based aflatoxin B_1_ suppressor and antifungal agent over the conventionally used fungicides and synthetic preservatives is irrefutable. Some of the findings validating this statement are summarized below in Table 3.

## 7. Safety Assessment

In the last decade, the internal use of *A. conyzoides* has been a matter of concern because of the distribution of some hepatotoxic constituents, pyrrolizidine alkaloids (PA), in the respective family, Asteraceae [138]. Notably, the occurrence or nonappearance of PAs fluctuates greatly from region to region. Some countries such as Belgium and Germany recommend the use of PAs within the limits approved to ensure the clinical safety of patients. Clinical studies are generally performed to validate ethno-medicinal claims and to establish the safety and efficacy of preparations in humans. Even though many researchers have assessed the plant and its constituents for a safer approach; there is a still dearth of the ample facts and figures that are required for an adequate inference about the dose required for a prompt action circumventing any toxicity issue. Nonetheless, these studies deliver a quite resilient source with which to trace future investigations.

Formerly, when the toxicity-related investigations started for precocene II, this chemical constituent was prepared in the lab. Stephen Hsia et al. [139] investigated the hepatotoxic effect of chemically synthesized precocene II in male SD rats and elucidated the mechanism(s) of precocene-induced pathological lesions. Acute i.p. dosing introduced severe necrosis of parenchymal cells of hepatic centrolobular regions, and intermediate metabolites formed in the liver were suggested as the responsible factor for this hepatotoxicity. Later in 1982, the same group assessed precocene-II-related nephrotoxicity in SD rats when administered via i.p. route: multiple (100 mg/kg body weight per day for 5 days) or single doses (200/300 mg/kg body weight) [140]. Investigations specified elevated BUN (blood urea nitrogen) levels, blood congestion in the glomeruli capillaries, tubular cell deterioration, and tubular cell restoration, reflecting the possibility of nephrotoxicity after a high dose and long exposure to the compound.

After a significant period, when the studies were resumed with the precocene II isolated from *A. conyzoides*, the observations were encouraging as compared with the synthesized one. In an investigation conducted by Moura et al. [141], results observed with lower doses of precocene II were quite impressive and safe. The study demonstrated the anti-inflammatory activity of *A. conyzoides* hydro alcoholic leaves extract for sub-acute (cotton pellet-induced granuloma) and chronic (formaldehyde-induced arthritis) inflammation models along with the evaluation of the occurrence or nonappearance of toxicity with prolonged use of this extract. For 90 days oral doses of 250 or 500 mg/kg body wt. were given to rats daily, and blood samples were collected and analyzed for hematological and biochemical parameters. The outcomes indicated that there was a reduction of 38.7% (*p* < 0.05) in cotton-pellet granuloma in the rats in the group treated with the extract at a dose of 250 mg/kg body wt., orally. Moreover, the plant extract significantly (*p* < 0.05) reduced the growth of paw edema induced chronically with no treatment-linked anomalies in either of the measured parameters. Although, in comparison with the control group, a 30.2% (*p* < 0.05) reduction of SGPT activity was observed in the rats group treated orally with 500 mg/kg body wt. The outcomes from the study justified the anti-inflammatory ability of *A. conyzoides* extract with no signs of apparent hepatotoxicity. Later, Igboasoiyi et al. [142] estimated the LD50 value and detrimental effects of the ethanolic extract of *A. conyzoides* on rats when administered the extract orally for 28 days daily at different concentrations of 500 and 1000 mg/kg. When evaluated, serum levels of all the enzymes and biomolecules assessed, such as aspartate and alanine aminotransferases (ASAT and ALAT), amylase, alkaline phosphatases, creatinine, total proteins, glucose, free fatty acid (FFA), low and very low-density lipoproteins, were not found to be affected significantly except for the high-density lipoproteins and cholesterol, whose levels reduced by a considerable range. In the interim, the mean lethal dose was calculated as 10.1 g/kg, and the results of the experiment did not claim any sort of toxicity on any organ, such as bone, kidney, pancreas, and liver, when used over a period of about one month, thus appearing safe. From these studies, ethanolic and hydroalcoholic extracts of the plant seemed to be safe as the results of sub-acute toxicity tests were fairly promising for these extracts.

Adebayo et al. [143] performed studies with higher doses along with doses lower than those studied previously and evaluated the association between the plant and isolated precocene II against hepatotoxicity risk using Sprague Dawley rats. Four groups—A, B, C, and D of rats—were administered with 0.25% CMC-Na per kg, 500, 1000, and 1500 mg/kg body wt. of ethanolic extract, respectively. Two indices of toxicity, hematological and biochemical, were measured by collecting the blood samples from anesthetized rats, whereas for the histopathological assays, liver, spleen, and kidney were dissected. Creatine kinase, aspartate amino transferase, alkaline phosphatase, and lactate dehydrogenase were considerably reduced in the C and D groups, whilst mean platelet volume, white blood cell count, and percentage of platelet distribution width were found to be significantly (*p* < 0.05) intensified. Furthermore, the histopathological studies showed an upturn in liver and spleen weight, along with several levels of hepatocellular necrosis in all the treated sets. In inference, the leaf extract of *A. conyzoides* was found accountable for altering the various biomarkers of skeletal and cardiac muscle disorders to a significant magnitude and certainly could be used in managing related diseases, whereas higher doses could encourage liver cell injury. In another study, Adebayo et al. [144] probed the isolation and purification in conjunction with the toxicological impact of the isolated compound precocene II, responsible for the antifungal property of the plant. Various procedures, such as column chromatography, certain purification steps, ^1^H-, ^13^C-, DEPT-NMR, and MS spectral methods were performed to obtain precocene from petroleum ether fractions of *A. conyzoides* L. Three groups—A, B, and C; eight rats in each group—received 0.25% CMC-Na, precocene II, 25 and 50 mg/kg body wt., respectively, through gastric intubation for 11 days. Animals were dissected on the 12th day and were evaluated for hematological, biochemical, and histopathological indices of toxicity. In conclusion, the constituent was evincing a hypoglycemic property and various hemopoietic values, such as red blood cell (RBC), mean corpuscular hemoglobin count (MCHC), platelet crit (PCT), and white blood cell (WBC) count, but no significant damage to the spleen, liver, or kidney tissues was found. Consequently, the investigations performed by Adebayo and group confirmed the safety of using *Ageratum* for various ethno-medicinal claims. However, treatment doses and duration still need to be determined.

Diallo et al. [145] explored the sub-chronic and acute toxicity associated with *A. conyzoides* in Wistar rats and suggested hydroalcoholic leaves extract as relatively safer. For the acute test, the rats were observed 1 h after the administration of a limited test dose of 5000 mg/kg daily for 14 days, while for evaluating the sub-chronic toxicity, oral doses of 1000 and 500 mg/kg body wt. were administered daily for 28 days. No signs of toxicity or were observed for acute dosing, nor was there any mortality. For chronic toxicity, relative organ weights and various hematological and biochemical parameters were assessed, in which no form of toxicity or any related abnormalities were observed. However, an increase in liver size was significant (*p* < 0.05) in the group receiving 1000 mg/kg, and urea was significantly (*p* < 0.05) lower in the group receiving 500 mg/kg of *A. conyzoides* extract. After this, Diallo et al. [146] administered *A. conyzoides* hydroalcoholic extract in late pregnant female rats; the results showed evidence of fetal toxicity, possibly caused by pyrrolizidine alkaloids-induced oxidative stress. Although all the female rats survived throughout the study, no visible symptoms of treatment were observed. Moreira et al. and Nweze and Obiwulu [71,147] observed a similar safety pattern as there were not any signs of toxicity reported in acute toxicity tests while they were investigating the plant for its anti-inflammatory and anti-coccidial properties, respectively. Recently, Adesanwo et al. [148] performed an acute toxicity test with varying concentrations between 10 mg/kg to 3000 mg/kg of methanolic extract of *Ageratum* administered in mice intraperitoneally. Extracts with concentrations of 10–1000 mg/kg appeared safe, but at concentrations above 1600 mg/kg, the methanolic extract became deadly. Additionally, the plant has attracted considerable attention because of its potential in minimizing the hepatic damage brought by stress or chemical exposure. Studies by Verma et al. [149] showed that *n*-hexane and acetone extracts of *A. conyzoides* repaired hepatic damage by restoring the normal limits of hepatic damage markers such as albumin, alanine transaminase (ALT), gamma-glutamyl transferase (GGT), lactate dehydrogenase (LDH-P), and conjugated and unconjugated bilirubin levels that were disturbed by acetaminophen introduction. A study performed by Ola Davies et al. [150] was in agreement; the study indicated the potential of *Ageratum* ethanolic leaf extract in protecting against sodium arsenite-induced toxicities.

Without a doubt, toxicological safety assessments are crucial prior to the clinical application of natural products as well as for their isolated compounds. Altogether, the toxicity studies conducted on the extracts, fractions, and isolated compounds from *A. conyzoides* appeared safe within a dosing fraction.

## 8. Future Research Prospective

Although, the *A. conyzoides* plant has proved its potential in various investigations carried out in diverse corners of the world, it is still unrecognized and ignored at the commercial level. Many reasons seem to be responsible for this neglect, which are summarized here along with possible ways that this noxious weed could be commercialized as a helpful and potentially profitable ethno-medical product.

The medicinal applications and toxicity profile of the *A. conyzoides* plant have not been studied well and still need to be investigated vigilantly by using advanced strategies. To fill in the remaining lacunae in the current understanding of the herb, long-term outcome studies with varying doses should be designed by considering a well-selected assortment of fungal strains to test, including fungi collected from different sources, such as from abiotic and biotic environments, clinical samples, healthy stocks, dead animals, food, and stored food samples so that the exact potential of the *A. conyzoides* plant can be assessed in terms of both quality and quantity. Hence, proper screening of the plant is required to draw any conclusion about its safety. Any use of *A. conyzoides* for long durations need to be managed carefully.

Additional studies also need to ascertain the appropriate dosage for human use and field trials need to explore the use of the plant as a natural fungicide with both a novel mechanism and high potency. Effective cooperation of microbiologists, clinical investigators, pharmacologists, and apothecaries should anticipate the development of *A. conyzoides* as a commercial botanical and fungicide by using the plant or the isolated compounds from the plant. Further, for measuring its quantifiable structure, activity–relationship studies should be carried out to explore new and improved products from the plant.

**Figure 5:**
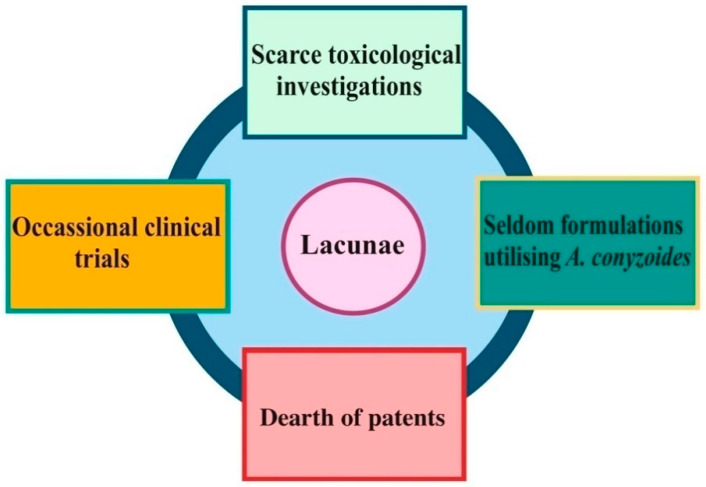
Lacunae observed in the clinical establishment of *Ageratum conyzoides.*

Various herbal or polyherbal dosages and formulations of *A. conyzoides* such as gels, emulsified concentrate, and nanoemulgel have shown improved wound healing, anti-inflammatory, and antimicrobial activity [151,152,153,154,155]. Still, there has not been ample progress in the context of formulation development by utilizing either the whole *A. conyzoides* L. plant or its extracts. Therefore, this could be a good way to innovate several dosage forms by investing more in progressive technology to enhance the commercial value of this invasive plant. There is a dearth of patents that have been filed and approved for *Ageratum* spp. formulations. China has patented a *Candida* (*C. albicans*, *C. tropicalis*, *C. krusei*) resistant medicine for treating deep and systemic fungal infections containing pogostone screened from various contents of *Ageratum* oil owing to its relatively higher bacteriostatic properties [156]. A herbal formulation for the treatment of several hair problems has also been patented based on this species [157]. Application of alien invasive plant *A. conyzoides* L. in control of golden apple snail has been patented based on the claim that a dry powder of the plant can kill *Pomacea canaliculate* [158]. Moreover, world governments should regulate the use of harmful biocides more strictly to encourage the development of safer and economic natural substitutes for fungi management. To establish a clinical role for this medicinally important herb, the hitches mentioned in the preceding paragraphs need to be removed (Figure 5).

The current scenario for the *A. conyzoides* plant is both inspiring and promising. South China is gaining economic and ecological benefits by incorporating the plant cautiously in its agro-ecosystem. In India, this plant is a constituent of an ayurvedic preparation “Arshonil Ointment” by “Nupol Ayurveda”, and it is used for rectal prolapse, itching anus, and anal fissure [159]. Herbalists of Kumansi, Ghana, are using leaves of this medicinal plant in a marketed antimalarial herbal product thatis registered with the Ghanaian FDA, bearing the code FDB/HD 12-9121 [160]. Among the Brazillian population, medicinal *A. conyzoides* tea is quite popular for its supposed anti-inflammatory and analgesic attributes, and it is included in the official herbal drugs list, created by the Brazilian Health Surveillance Agency, Anvisa; this means that the natural drug is approved for marketing without a medical prescription [38]. Significantly, this country considers *Ageratum* as a species that needs conservation and germplasm collection [161]. Moreover, some trifling companies of pharmaceutical world in Brazil have already introduced the plant as a fresh source of material for phytochemicals. Eventually, the demand will hasten with time, warranting more advanced research to develop *A. conyzoides* for both remedial and agrarian use.

## 9. Conclusions

The high cost and limited availability of antifungal drugs (polyenes, imidazoles, and azoles), rapid multidrug-resistant fungal spread, and the resistance that accompanies conventional synthetic fungicides all justify the need to develop novel strategies and alternative ways to curtail these issues, such as with plant extracts. Recently, researchers around the globe have devoted a lot of their work to exploiting certain selected plants and their metabolites with hopes of discovering new therapeutics that can thwart these resistant mechanisms and that are easily available in nature. Moreover, essential oils obtained from these plants, having antifungal properties, have more or less been categorized as approved flavors or food additives by the United States Food and Drug Administration under the category “generally recognized as safe”. Amongst these natural resources, *A. conyzoides* has been privileged enough to draw the attention of scientists worldwide, and it seems to carry the potential of introducing a fresh approach into contemporary medicine. Owing to its encroachment upon an outsized region, this plant may deliver an economical source of an appreciated fungi-toxicant. Affirmative clinical introductory assays conducted on the plant have undoubtedly validated its great commercial importance in some tropical nations. Nevertheless, more clinical studies are needed to launch the plant at commercial scale and provide a range of novel valuable natural antifungals.

Consequently, awareness about this plant and its therapeutic importance needs to be built among the pharmacologists and researchers of the world by filling in the immense space of limited information about *A. conyzoides*. This breach can only be addressed by creating interest among scientists through the writing of updated reviews and by carrying out investigations on various aspects of *A. conyzoides*.

## Figures and Tables

**Table 1:** Traditional uses of *Ageratum conyzoides* L.

Country	Traditional Uses	Plant Part/Medicinal Preparation(s)/Doses	Reference(s)
Nigeria	Diarrhea	Plant decoction of leaves and aerial branches of *A. conyzoides* L. and stem bark of *Annona senegalensis* Pers. (Annonaceae) is taken thrice a day	[39]
	Diabetes	Whole plant/macerated with two other herbs—*Stachytarpheta indica* Vahl. (Verbanaceae) and *Sorghum guinensis* (Linn) Moench (Poacea)—is consumed twice a day	
	Earache	Warm leaves exudate squeezed as ear drops	
	Eaten by Igbo communities	Part of “olulu-ogwai” soup	[40]
Brazil	Diarrhea, menstrual cramps, rheumatism, and arthritis	Aerial parts (dried or fresh, externally and internally as infusions or tinctures) and in medicinal teas	[41]
	Analgesic and anti-inflammatory		[42]
Cameroon	Syphilis condition	Leaves (mixed with other herbs)	[43]
	Craw-craw (itching skin disease)	NS	[44]
Ghana	Eyetroubles	Rub and squeeze (Topical)	[45]
	Antifungal and antibacterial	NS	[46]
	To augment hair growth and in constipation (as an enema)	Children’s eyebrows scrubbed with charcoal punched young stems of plant	[47]
Western Nepal	Wounds and cuts	Juice of leaves	[48]
Gabon	In helminthiasis	Decoction of leaves	[49]
	and malaria	NS	[50]
Congo	Treating chronic pain, analgesic, antimicrobial, and anti-inflammatory	Leaf extract	[51]
African countries	To cure contagious and psychological diseases, diabetes, snake bite antidote	NS	[52]
	Pneumonia, wounds, and burns	NS	[30]
	Cure scabies, anti-asthmatic, dyspnea, antispasmodic, and hemostatic effects	NS	[44]
Tanzania	Stomachache	Leaves are chewed	[53]
	Wound healing	Pounded fresh leaves	
	Cough and chest congestion	Roots	
Indonesia	Against fungal infection	NS	[54]
	Wounds, eczema, ulcers and in bacterial infections	NS	[55]
India	To stop bleeding	Leaf extract	[56]
	Anthelmintic and wound healing	Stem and Leaf	[57]
	Wounds and cuts	Leaf paste	[58,59]
	Eye discharge and leprosy	Oil lotion	[60]

* NS; Not stated.

**Table 2:** Antifungal activities by main constituents extracted from *Ageratum conyzoides.*

Chemical Constituent	Fungal Strain Investigated	Inference/Mechanism of Action	Reference(s)
Precocene II	*Phomopsis theae*, *Botryodiplodia theobromae*, *Rhizoctonia solani*, *Sclerotium rolfsii*, and *Fusarium species*	*R. solani* and *S. rolfsii* sclerotia were completely suppressed by 150 ppm precocene II. Sub-cultures of inhibited strains on precocene II-free media refurbished fungal growth, confirming the fungicidal activity of precocene II isolated.	[91]
Precocene II	* Aspergillus flavus *	Fungal growth was restricted to different extents, and aflatoxin production was inhibited completely above concentrations of 0.10 µg/mL. Transmission electron microscopy (TEM) showed ultra-structural alterations, prominently in endomembrane system, largely affecting the mitochondria. Surrounding fibrils were also reported as degraded.	[92]
Precocene II	* Fusarium graminearum *	Superoxide level was augmented in mitochondria, and eventually, trichothecene production was inhibited in *Fusarium graminearum* after treating with precocene II.	[93]
Precocene II	* Aspergillus flavus *	Among the three oils investigated (5.0 μL; from 3 different locations), the oils with more precocene II concentration inhibited the fungal growth effectively.	[94]
Eugenol	* Botrytis cinerea *	Various eugenol concentrations (0, 25, 50, 100, 150, and 200 μg/mL) inhibited *B*. *cinerea* growth in a concentration-dependent way. Eugenol EC_50_ reported was 38.6 μg/mL on mycelial radial growth of *B*. *cinerea.* In light and scanning electron microscopy, morphological changes—namely, cytoplasmic coagulation, hyphal shrivelling and vacuolation—were revealed after exposure to eugenol. However, eugenol did not show any activity against conidia germination.	[95]
Eugenol	*Candida albicans*, *C. krusei*, and *C. glabrata*	At sub-MICs (6.25–100 mM), eugenol inhibited the formation of germ tube by *C. albicans* completely and was found highly toxic to all fungal strains within 2.5 h of exposure. The results by SEM confirmed eugenol-induced cellular deformity.	[96]
Eugenol	* Fusarium oxysporum * MTCC 284, *F. moniliforme* NCIM1100, *Mucor* *sp*., *Aspergillus* *sp*., *Microsporum gypseum*, and *Trichophyton rubrum*	Order of sensitivity: *Mucor* *sp*. > *M. gypseum* > *F. monoliforme* > *T. rubrum* > *Aspergillus* *sp*. > *F. oxysporum* For the tested strains, MIC was reported as 9–12 µL/mL. Eugenol caused distortion and shrinkage on spores of *M. gypseum* and *Mucor* sp.	[97]
Eugenol and β-caryophyllene	53 human pathogenic yeasts (All candida species)	Bud oil, 10 mg per disc, was reported effective against all the fungal strains investigated.	[98]
Eugenol	*Zygosaccharomyces rouxii*	MIC (minimum inhibitory concentrations) and MIF (minimum fungicidal concentrations) for eugenol was reported as 0.4 μL/mL and 0.8 μL/mL, respectively. SEM presented wrinkles and torn cell surfaces upon eugenol treatment. Additionally, the permeability studies revealed that eugenol induced abolishment of cell membrane permeability, leading to electrolytes loss and ultimately *Z. rouxii* death.	[99]
β-caryophyllene	*F. solani*, *Aspergillus fumigatus*, *A. parasiticum*, and *A. niger*	β-caryophyllene demonstrated a rapid and efficient fungicidal action within 4–8 h and 2–4 h for *A. niger* and *F. solani*, respectively. MIC and MFC both values were reported higher for β-caryophyllene than essential oil (evaluated for 2.0 to 0.015 mg/mL concentrations), signifying the synergistic effect among the oil components.	[100]
β-caryophyllene	* Trichoderma reesei * and *A. niger*	β-caryophyllene was observed with a more pronounced antifungal effect than kanamycin, standard reference. MIC reported was 6 ± 0.8 μM and 4 ± 0.7 μM for *A. niger* and *Trichoderma reesei*, respectively.	[101]
Coumarin	*Candida albicans*	Coumarin showed a clear inhibition zone up to 72 h as compared with 24 h of miconazole nitrate. Among various coumarin concentrations tested (31.25, 62.5, 125, 250, 500, 1000 µg mL^−1^ in dichloromethane), MIC reported was 125 μg/mL. Scanning electron microscope (SEM) and TEM analytic exploration observed that the compound damaged the fungal cells by pores development in the cell wall, allowing escape and necrosis of cytoplasmic content leading to death.	[102]
Coumarin	*Candida albicans*	Different coumarin concentrations, i.e., 0.5, 1.0, and 2.0 mg/mL, significantly inhibited fungal growth in a dose-dependent manner. This constituent induced a sequence of apoptotic features such as phosphatidylserine (PS) externalization, fragmenting DNA, and condensing nucleus. Coumarin treatment was also reported to alter the mitochondrial morphology.	[103]

**Table 3:** Evidence of the activity of essential oil/different extracts from *Ageratum conyzoides* against important fungal strains.

Plant Part Used (Location)	Type of Extract (Conc.)	Fungal Strains Investigated	Inference	References
Leaves (Nigeria)	Aqueous extracts (15, 30, 45, and 60% concentrations)	*Pseudoperenospora cubensis* causes downy mildew disease, muskmelon	Leaf extract inhibited radial growth and conidia germination significantly in comparison with the control. However, sporulation of *P. cubensis* was unaffected when treated with the extract.	[119]
Leaves (Côte d’Ivoire)	Aqueous total extract, ethanolic fractions, and aqueous residual fractions (50 to 0.097 mg/mL)	*Trichophyton mentagrophytes*	Among the three extracts investigated, 70% ethanolic fraction of leaves showed higher level activity against the fungal colony in comparison with the other extracts. A minimum concentration fungicide (MCF) of 1.56 mg/mL and IC_50_ value of 0.29 mg/mL was observed for this fraction.	[120]
Whole parts (Indonesia)	Methanol crude extract (0.1, 1.0, 2.5, and 5.0%)	*Puccinia arachidis Speg* causes rust disease in peanut leaves	At concentrations of 2.5% and 5.0%, *Ageratum* extracts protected the crop loss of 67.5% and 63.5%, respectively, by significantly inhibiting the intensity of rust disease.	[121]
Leaves (India)	Aqueous extracts (5,10,15, and 20%)	*Aspergillus niger* causes pineapple fruit rot pathogen	*Ageratum* was found to be less effective even after 48 h of 20% extract treatment; 80% mycelial growth was retained by the fungal strain tested.	[122]
Leaves (India)	Petroleum ether and methanolic extracts (1 mL of dil. Plant extract (20 mg/mL) mixed with 19 mL potato dextrose agar)	*Geotrichum candidum*, *F.oxysporum*, and *A. niger*, cause decay of *Dioscorea alata* L. (yam) tubers	Pet. ether extract of the plant significantly inhibited the growth of all the fungi examined. Extract was found better even than the synthetic fungicides (Indofil M-45, Blitox-50, and Mancozeb except Dhanustin) when compared for mycelial growth percentage inhibition.	[123]
Aerial parts (leaves and stem) (Cameroon)	Aqueous extracts (5,10, 15, 20 mg/mL) and ethanolic extracts (1.25, 2.5, 5, 10 mg/mL)	*Botryodiplodia theobromae* and *Colletotrichum gloeosporioides* cause pod rot disease of Cocoa, *Theobroma cacao*	For ethanolic extract, complete (100%) growth inhibition of both the fungi was reported at 10 mg/mL concentration. For aqueous extracts, a concentration of 20 mg/mL completely inhibited the *B. theobromae* growth whilst *C. gloeosporioides* growth was suppressed up to 78%.	[124]
Root, shoot, and leaf (Pakistan)	Essential oils, aqueous extracts, and dichloromethane (DCM) fraction (Stock Solution: 20% *w*/*v*; for antifungal assay: 1–4% concentrations in distilled water)	*Drechslera australiensis* and *D. holmii* cause brown spot, leaf blight, root rot, and crown rot of crops	The order of activity against the fungal growth was observed as, essential oil > dichloromethane extract > aqueous extract. Dichloromethane fraction of shoots (4%) exhibited highest biomass depression of 91% and 92% in *D. holmii* and *D. australiensis*, respectively. Aqueous extracts at lower concentrations found to arrest mycelial growth, while the growth was being favored at higher concentrations. Essential oil was found capable of arresting mycelial growth at all the concentrations i.e., 1–4%.	[125]
Leaves (Brazil)	Concentrated hexane extract (25, 50, and 100 mg/mL dilutions with dichloromethane); 1 mL extract was mixed with 9 mL culture medium)	*Leucoagaricus gongylophorus* (Singer) Möller (symbiotic for leaf cutting ants)	Extracts of *A. conyzoides* unveiled 81, 93, and 100% decline in the fungal biomass at various concentrations of 25, 50, and 100 mg/mL. Consequently, the plant may be used further in controlling the leaf-cutting ants that live in symbiosis with the inspected fungus.	[126]
Leaves (India)	Essential oil (hydro–distilled) (100 µL)	*Alterneria alternata*, *Mucor hiemalis*, *Helminthosporium solani*, *Humicola grisea*, and *Botrytis cinerea*	Study revealed the potential of *Ageratum* as a part of integrated pest management system. Oil effectively restricted the growth of two phytopathogenic fungi, *A. alternata* and *H. solani*, out of five tested fungal strains.	[127]
Leaves (Brazil)	Essential oil (10, 15, 30, and 50 µL)	*Aspergillus flavus*, aflatoxin B_1_ production in real food systems (corn and soybean)	Precocene I (96.53%) and precocene II (2.40%) were the key constituents reported in the oil. 90% aflatoxin production inhibition by using the volumes of 48.5 and 14.1 μL was observed for corn and soybeans, respectively.	[128]
Leaves (India)	Hydroalcoholic extract (5, 10, 15, and 20%)	*Pediculus humanus capitis*, head lice	After exposure to *Ageratum* extract, mortality % age of head louse was reportedly comparable to the marketed pediculicidal formulation, mediker. Safety study: No oedema or erythema caused when applied topically on the rabbit’s skin for safety evaluation.	[62]
Whole plant (Cameroon)	Essential oil, cold water, hot water, and ethanol extract (100–5000 ppm)	*Phytophthora infestans* pathogen, late blight disease (potato and tomato)	Highest mycelial inhibitory potential was demonstrated with essential oil, followed by the ethanolic extract. Fungicidal activity for ethanolic extract was observed at a concentration of 5000 ppm.	[129]
Whole plant (India)	Methanol, ethyl acetate, benzene chloroform, and acetone extracts (800 µL broth +100 µL plant solvent extract +100 µL fungal suspension culture)	*Alternaria SPP*, a phytopathogenic fungus	The fungus examined was observed to be highly sensitive toward the chloroform and methanolic extracts, with a minimum inhibitory concentration of 3.125 × 10^−5^ μL/mL and 6.25 × 10^−4^ μL/mL.	[130]
Aerial parts (India)	Essential oil (hydro–distilled) (10, 25, 50, 75, and 90%)	*Phoma medicaginis*, *Sclerotium rolfsii*, *Rhizoctonia solani*, *Fusarium solani*, *F. oxysporum*, and *Alternaria brassicaceae*	The essential oil (10 to 90%) exhibited a varied zone of inhibition against R. solani (5.00 to 10.00 mm), *S. rolfsii* (12.67 to 24.89 mm), *F. solani* (6.00 to 9.00 mm), and *F. oxysporum* (4.00 to 10.00 mm). Although *Ageratum* oil at 10% concentration did not inhibit the growth of *Alternaria brassicaceae* and *Phoma medicaginis*, afterward its activity was observed to be concentration dependent.	[131]
Leaves (India)	Essential oil (hydro–distilled) (0.08–1.2 µL/mL)	Toxigenic strain, *Aspergillus flavus* (Saktiman 3NSt) Storage fungi, *Aspergillus niger*, *A. terreus*, *A. fumigatus*, *Alternaria alternata*, *Cladosporium cladosporioides*, *Fusarium roseum*, *Curvularia lunata*, *Trichoderma viride*, and *Penicillium italicum*	Study confirmed the broad fungi static spectrum owned by the oil. At a concentration of 1.0 μL /mL, oil was toxic against the toxigenic strain tested. Invivo evaluation when carried out by fumigating the stored wheat samples with oil showed a remarkable (>80%) protection of sample against food borne fungi and presented it as a better natural food preservative over harmful synthetic preservatives.	[132]
Shoot and root extracts (Pakistan)	Aqueous extract (2, 4, and 6%)	*Macrophomina phaseolina* (*Tassi*) *Goid.* cause charcoal rot disease of sunflower (*Helianthus annus* L.)	At varied concentrations (2–6%), *A. conyzoides* showed a dissimilar pattern of percentage reduction in fungal biomass production for root (49–71%) and shoot extracts (48–69%). 4% shoot extract was observed as most affective.	[84]
Shoots (Sri Lanka)	Sequentially extracted with *n*-hexane, dichloromethane, and methanol (crude extract: 0.1%; *n*-hexane extract: 200, 500, 3000 ppm; precocene II: 10 ppm–500 ppm)	*Phomopsis theae*, *Botryodiplodia theobromae*, *Rhizoctonia solani*, *Sclerotium rolfsii*, and *Fusarium species*	MIC communicated for *R.solani* and *S. rolfsii* was 500 ppm of crude *n*-hexane extract whilst the response against other assayed fungi varied in a dose-dependent manner. Fungicidal activity of the other two organic solvent extracts was not significant. Confirmed the fungicidal activity of precocene II isolated.	[91]
Whole plant (China)	*A. conyzoides* intercropped with citrus orchards	*Pythium aphanidermatum*, *Phytophthora citrophthora*, and *Fusarium solani*	The inter-cropped plants of *A. conyzoides* did not allow other weeds to grow by covering the orchard ground and also stifled the growth of soil pathogenic fungi in propinquity. In greenhouse, allelochemicals released by the plant at higher concentrations (>300 μg/g) could slightly inhibit the growth of orchid seedlings. However, the trend was not apparently followed in citrus orchard intercropped.	[90]
Root and shoot (Pakistan)	Aqueous extract (5, 10, 15, and 20%)	*Aspergillus niger* Van Tieghem *A. fumigatus Fresenius* *A. Nidulans Eidam*	*A. Fumigatus* growth was significantly clogged in a concentration-dependent manner (Max. at 20%) when exposed to the extracts, whilst the biocidal effect on other strains was not found ample.	[133]
Shoots (Sri Lanka)	Aqueous extracts (3 mL plant extract with 20 mL potato Dextrose agar)	*Pestalotiopsis theae*, *Aspergillus niger*, *Botryodiplodia theobromae*, and *Rhizoctonia solani*	Except *B. theobromae*, mycelial growth for rest of the fungi was suppressed by at least 70%, after 3 days of incubation. Fungal inhibitory factor was found to retain its activity even at higher temperatures of 1210 °C.	[134]
Leaves (Brazil)	Crude extract (1, 5,10, 15, 20, 25, and 50%); essential oil (20, 40, 60, 100, 200, 500, and 1000 µL)	*Didymella bryoniae* (Auersw.) causes gummy stem blight (affects melon crop)	Inhibition of fungal mycelial growth and spore germination varied with various concentrations of crude extract used. 20 μL and 100 μL of oil completely inhibited the mycelial growth and spore germination, respectively.	[135]
Leaves (India)	Essential oil (0.1, 0.2, and 0.3%)	*Penicillium italicum* causes blue mold rot of mandarins	Among the plants of 30 species screened, vapors from *A. conyzoides* leaf oil unveiled the highest inhibitory potential (MIC, 0.2%) against *Penicillium italicum.* Broad fungistatic continuum was shown by *Ageratum* oil at 0.3% concentration, attributable to inhibition of 32 storing fungi out of 35 tested. In vivo studies, confirmed no damaging effects on the quality of treated fruits.	[136]
Leaves (India)	Essential oil (250 and 500 µL/L)	*Helminthosporium oryzae*	At minimum inhibitory concentration, 250 μg/L, oil inhibited the mycelial growth entirely. When in vivo trials were performed, the oil was found competent enough to check the entrance of leaf spot disease of paddy and was nontoxic to the crop.	[137]

## Data Availability

No new data were created or analyzed in this study. Data sharing is not applicable to this article.
